# Nortropane alkaloids as pharmacological chaperones in the rescue of equine adipose-derived mesenchymal stromal stem cells affected by metabolic syndrome through mitochondrial potentiation, endoplasmic reticulum stress mitigation and insulin resistance alleviation

**DOI:** 10.1186/s13287-019-1292-z

**Published:** 2019-06-18

**Authors:** Lynda Bourebaba, Fatiha Bedjou, Michael Röcken, Krzysztof Marycz

**Affiliations:** 1 Department of Experimental Biology, Faculty of Biology and Animal Science, Wrocław University of Environmental and Life Sciences, Norwida 27B, 50-375 Wrocław, Poland; 2 Faculty of Veterinary Medicine, Equine Clinic - Equine Surgery, Justus-Liebig-University, 35392 Gießen, Germany; 3 Laboratoire de Biotechnologies végétales et d’Ethnobotanique, Faculté des Sciences de la Nature et de la Vie, Université de Bejaia, 06000 Bejaia, Algeria; 4 International Institute of Translational Medicine, Jesionowa, 11, Malin, 55-114 Wisznia Mała, Poland

**Keywords:** EMS, ASCs, *Hyoscyamus albus*, Calystegines, Iminosugars, Insulin resistance

## Abstract

**Objectives:**

Equine metabolic syndrome (EMS) refers to a cluster of associated abnormalities and metabolic disorders, including insulin resistance and adiposity. The numerous biological properties of mesenchymal stem cells (MSCs), including self-renewal and multipotency, have been the subject of many in-depth studies, for the management of EMS; however, it has been shown that this cell type may be affected by the condition, impairing thus seriously their therapeutic potential. Therefore, an attempt to rescue EMS adipose-derived stem cells (ASCs) with calystegines (polyhydroxylated alkaloids) that are endowed with strong antioxidant and antidiabetic abilities was performed.

**Methods:**

ASCs isolated from EMS horses were subsequently treated with various concentrations of total calystegines. Different parameters were then assessed using flow cytometry, confocal as well as SE microscopy, and RT-qPCR.

**Results:**

Our results clearly demonstrated that calystegines could improve EqASC viability and proliferation and significantly reduce apoptosis, via improvement of mitochondrial potentiation and functionality, regulation of pro- and anti-apoptotic pathways, and suppression of ER stress. Furthermore, nortropanes positively upregulated *GLUT4* and *IRS* transcripts, indicating a possible sensitizing or mimetic effect to insulin. Most interesting finding in this investigation lies in the modulatory effect of autophagy, a process that allows the maintenance of cellular homeostasis; calystegines acted as pharmacological chaperones to promote cell survival.

**Conclusion:**

Obtained data open new perspectives in the development of new drugs, which may improve the metabolic dynamics of cells challenged by MS.

## Introduction

Metabolic syndrome (MS) which represents a common and growing health problem in horses was first described as hypothyroidism, peripheral Cushing’s disease, or pre-Cushing’s syndrome [1]. More recently, equine metabolic syndrome (EMS) has been linked to three key abnormalities, namely generalized or regional adiposity, insulin resistance, and laminitis. Most affected equids systematically combine these three conditions; however, some lean phenotypes could also be observed [2]. Although some equids with MS may appear with a normal body mass index, obesity is observed in the majority of cases. Regional adiposity is keenly characterized by the expansion of subcutaneous adipose tissue and fat accumulation in various parts of the body. Often associated with obesity, insulin resistance with hyperinsulinemia or inadequate glycemic and insulinemic responses is also one of the main clinical manifestations of the syndrome. Finally, inflammatory episodes associated with laminitis, whether clinical or subclinical, are closely related to the condition [3].

When obesity develops, adipose tissue adopts a distinctly inflammatory phenotype, characterized by increased gene expression and secretion of pro-inflammatory cytokines (such as TNFα, IL-1β, and IL-6) [4, 5]. In addition, obese adipose tissue usually displays an altered production of the adipokines leptin and adiponectin, which have systemic and tissue-level effects on metabolism and inflammation [6]. Insulin plays a central metabolic role in maintaining whole-body glucose homeostasis and promoting efficient glucose utilization in skeletal muscle, adipocytes, and liver [7]. However, when insulin levels become higher than expected relative to the level of glucose in the body, insulin resistance condition appears. Insulin resistance (IR) by definition refers to decreased sensitivity or responsiveness to insulin-mediated glucose disposal or inhibition of hepatic glucose production [8]. Mechanisms by which IR occurs mainly include breakdown of insulin-signaling mediators and downregulation of insulin receptors. However, the most common mechanisms observed during IR, involve alterations in post-receptor binding signaling pathways, associated with a decrease in autophosphorylation of β sub-units, and therefore decreased tyrosine kinase activity [9, 10].

Mesenchymal stem cells (MSCs) are since many years gaining popularity in equine veterinary medicine for regenerative purposes, not only because of their potential for differentiation but also because of their trophic, anti-inflammatory, and immunomodulatory abilities [11]. Indeed, adult mesenchymal stem cells (MSCs) alone, or in association with biological scaffolds, have attracted scientific and clinical interest because of their self-renewal ability and their multilineage differentiation potential, conferring them a high value in tissue engineering [12]. In addition, one of the peculiarities of adipose stromal cells (ASCs) certainly lies in the paracrine action that they can modulate through secretion of a wide range of growth factors, anti-apoptotic and anti-inflammatory mediators in membrane-derived vesicles (MVs). MVs are released by ASCs to extracellular space and, except for the fact that they deliver a plenty of growth factors (FGF, VEGF, and HGF), they improve cell to cell signaling, which seems to be fundamental in the process of regeneration [13]. MSCs have therefore been considered as a good therapeutic strategy for the management of type 2 diabetes mellitus. MSCs have thus previously demonstrated potent capacity to promote pancreatic islet beta cell regeneration, to preserve them from apoptosis and to reduce insulin resistance in peripheral tissues by modulating a microenvironment propitious to secretion of paracrine factors or extracellular matrix deposition; suggesting that mesenchymal stem cells would be a promising alternative in the treatment of MS [14]. Nonetheless, it has been previously demonstrated that endogenous repair system that ASCs constitute may be severely impaired by exposure to conditions constituting the diseases. In addition, the ability to administer autologous cell-based therapies has been impeded by a fall in MSC quantity and quality during disease states [13, 15]. In this respect, many studies have shown that ASCs isolated from horses affected by EMS were markedly characterized by increased oxidative stress, senescence, and apoptotic phenotype. Moreover, a high level of mitochondrial and ER stress caused by excessive ROS was also observed in the same cells [16–18]. Because pathological conditions encountered in various diseases such as MS can severely affect the healing and regenerative potential of MSCs, preconditioning with potentially therapeutic substances, such as natural plant extracts, is presumed to protect MSCs from injury and improve their application in regenerative medicine, further activating endogenous cellular machinery for regeneration [19].

Numerous herbal remedies have been used in traditional medicine for centuries in the treatment of a wide range of different pathologies. These are indeed currently considered as a promising alternative in the development of new pharmaceutical formulations, as they offer a plenty of bioactive substances such as polyphenols, essential oils, and alkaloids. Although the mechanisms of action of these plants and their respective extracts remain still unclear, the study of influences on proliferation, differentiation, and cytotoxicity of different herbal extracts on stem cells could be an alternative option to their rescue and the maintenance of the endogenous repair system that constitutes these cells [20]. *Hyoscyamus albus L.*, also called white henbane, is a Mediterranean plant belonging to the *Solanaceae* family, which is considered to be of great importance for human beings from medicinal and economic point of view. All species of the genus *Hyoscyamus* produce tropane alkaloids, namely hyoscyamine and scopolamine, which are renowned for their mydriatic, antispasmodic, anticholinergic, analgesic, and sedative properties [21]. More recently, a new group of polyhydroxylated nortropane alkaloids named calystegines has been isolated from different species of *Solanaceae* including *Hyoscyamus* [22]. The discovery of polyhydroxy alkaloids, otherwise known as iminosugars, raised an important interest in therapeutics because of their ability to inhibit different glycosidases, which confers them a plenty of possible biological activities. In this sense, several investigations have already demonstrated the antidiabetic, antihyperlipidimic, hypoglycaemic, antioxidant, and anti-inflammatory effect as well as pharmacological chaperone activity in Gaucher’s disease of these alkaloids, suggesting a possible use in the treatment of multifactorial pathologies such as EMS [23–25].

The present investigation aimed therefore to the exploration of the effects of total calystegines extracted from white henbane in the rescue of ASCs isolated from EMS horses, with the potential of reducing their resistance to insulin as well as promoting their viability in order to restore their subsequent physiological actions.

## Materials and methods

### Plant materials

Seeds used for calystegine isolation were collected from *Hyoscyamus albus* wild growing specimens in August 2016 from the area of Bouzguene, city of Tizi-Ouzou, Algeria (36° 37′ 0″ N 4° 28′ 47″ E). Seed samples were removed from the dried calyxes, dehydrated in a ventilated room (30 ± 3 °C) and then ground to a fine powder and stored in the dark before use.

### Chemicals

Solvents used for extraction and GC-MS analysis were from HPLC grad, and resins (Amrelite IR 120B, H^+^, Dowex 1X2, Cl^−^) were purchased from Sigma Aldrich (Barcelona, Spain). All other reagents used in the study were purchased from Sigma Aldrich (Taufkirchen, Germany) unless otherwise specified.

### Extraction and isolation of total calystegines

Total calystegines were extracted from *H. albus* seeds as previously described by Bourebaba et al. [23]. Briefly, powdered seeds (50 g) were first defatted three times using 250 ml petroleum ether prior to hydroalcoholic extraction. Crud extract was then prepared by homogenizing the defatted powder with 250 ml aqueous methanol (50/50; *v*/*v*), three times each during 24 h. Calystegines were after that isolated by applying the obtained dried extract to a cation exchange column (Amberlite IR 120B, H^+^ form). Non-binding contaminants were subsequently removed by washing the column with distilled water, and bound compounds including calystegines were eluted with 2 N NH_4_OH. The concentrated residue was then subjected to an anion exchange column (Dowex 1X2, Cl^−^ form) and eluted with distilled water to obtain a purified calystegine-rich extract.

### Gas chromatography–mass spectrometry analysis (GC-MS)

Total calystegines extract was characterized by means of a gas chromatography–mass spectrometry analysis according to the procedure described by Bourebaba et al. [24]. A trimethylsilyl trifluoroacetamide (MSTFA) derivatization step was conducted prior to chromatographic analysis, and a GCMS-QP2010 plus system (Shimadzu, Kyoto, Japan) equipped with a DB-5 ms column (30 m × 0.25 mm I.D. × 0.25 μm df, Quadrex Corporation, Woodbridge, CT) was employed for the characterization. Separation of extract compounds was performed according to the following oven temperature program: initial temperature was 100 °C and held for 5 min, then raised to 300 °C at 10 °C/min, and this value was maintained for 5 min. The injection volume was 0.5 μl in split mode (split ratio 1:10) with the injector temperature at 250 °C. Carrier gas was He at 36.5 cm/s. The MS detection parameters were interface temperature, 280 °C; ion source temperature, 250 °C; mass range, *m*/*z* 50–600; scan speed, 2500 amu/seg; and event time, 0.20 seg. The data collection and handling were performed using the GCMS solution (ver. 2.50SU3, Shimadzu) software.

### Equine EMS ASC isolation and cell culture

Adipose tissue samples were obtained from the tail base area of adult EMS and healthy horses, under local anesthesia induced by 2% lidocaine (Polfa S.A., Warsaw, Poland). All samples were extensively washed using Hanks’ balanced salt solution (HBSS) supplemented with 1% antibiotics for eventual microbial contamination. Tissues were then excised, finely minced using surgical scissors, digested in the presence of collagenase type I solution (0.1 mg/mL) for 40 min at 37 °C and 5% CO_2_, and centrifuged at 1200×*g*, 10 min. The remaining cell pellet was after that resuspended with Dulbecco’s modified Eagle’s medium (DMEM) containing 1000 mg/L glucose and supplemented with 5% of fetal bovine serum (FBS), and 1% of a penicillin and streptomycin (PS) solution in culture flasks. Cultures were maintained in a humidified CO_2_ incubator (37 °C, 5% CO2, 95% air atmosphere) and harvested every 3 days (80–90% of confluence) using a trypsin-EDTA solution (TrypLE Express, Life Technologies).

The cells used for experiments were passaged three times, and cellular purity was assessed using fluorescent-activated cell sorting technique (BD FACSCalibur, Becton Dickinson, Franklin Lakes, NJ, USA). ASC phenotyping was performed by flow cytometry analysis using fluorochrome-conjugated monoclonal antibodies (anti-CD105, Acris, Herford, Germany, SM1177PT; anti-CD45, Novus Biologicals, Littleton, CO, USA, NB1006590APC, anti-CD44, R&D Systems, Minneapolis, MN, USA, MAB5449, anti-CD90, ab225; Abcam, Cambridge, UK). Multipotency of isolated ASCs was confirmed by osteogenic, chondrogenic, and adipogenic differentiation of cells cultured in StemXVivo kits (R&D System). All abovementioned assays were extensively described previously [16–18].

Isolated cells expressed significant amounts of positive cell surface markers, namely CD90 and CD105, and were negative for CD45 and CD34, which excluded their hematopoietic origin. Moreover, the multipotent nature of ASCs was confirmed by positive results of differentiation into osteoblast, chondrocytes, or adipocytes in vitro [16–18].

### Alamar Blue viability assay

Cell proliferation was estimated using the resazurin-resorufin test (*TOX8*). Briefly, EMS_ASCs_ cells were seeded at a density of 8 × 10^3^ cells per well in 96-well plates. After cell attachment, calystegines were added to the cells at different concentrations and incubated for 72 h. Culture medium was then replaced with a 10% resazurin solution (Alamar Blue, Sigma Aldrich, Poznàn, Poland), and plates were incubated with dye for 2 h at 37 °C. Resazurin reduction was measured using a spectrometer (Spectrostar Nano; BMG Labtech, Ortenberg, Germany) at the specific wavelengths: 600 nm for resazurin and 690 nm as a background absorbance.

### Measurement of DNA synthesis

DNA synthesis was assessed using the BrdU Cell Proliferation ELISA Kit (Abcam, Cambridge, UK) according to the manufacturer’s instructions. Briefly, cells were pre-treated with various concentrations of calystegines, and then, BrdU was added to cell cultures after 48 h and left overnight at 37 °C to achieve 72-h incubation. Incorporation of 5-bromo-2-deoxyuridine (BrdU) into cellular DNA was determined by incubating fixed cells with anti-BrdU monoclonal antibody, and Goat anti-mouse IgG conjugated with horseradish peroxidase (HRP) as a secondary antibody. HRP substrate degradation was measured with a spectrophotometer plate reader (Spectrostar Nano; BMG Labtech, Ortenberg, Germany) at a wavelength of 450/550 nm.

### Evaluation of cell morphology

Cell morphology was evaluated by confocal microscopy (Observer Z1 Confocal Spinning Disc V.2 Zeiss with live imaging chamber) and scanning electron microscopy (SEM, Zeiss Evo LS 15). Briefly, calystegine-treated and control-untreated cells were fixed with 4% paraformaldehyde (PFA) at room temperature for 45 min. Samples were after that rinsed with Hank’s balanced salt solution (HBSS), then cell membranes were permeabilized using 0.1% Triton X-100 solution for 15 min at room temperature. Actin filaments were stained using atto-590-labeled phalloidin at dilution 1:800 with HBSS for 40 min, in the dark at room temperature. Nuclei were imaged by the mean of diamidino-2-phenylindole (DAPI), using the ProLong™ Diamond Antifade Mountant with DAPI (Invitrogen™, Poland). Mitochondria were stained using the Mito Red fluorescence dye diluted at 1:1000 in medium, and incubated for 30 min at 37 °C in a CO_2_ incubator, prior to PFA fixation. All images were captured with a Canon PowerShot camera. Obtained photomicrographs were merged and analyzed using *ImageJ* software (Bethesda, MD, USA). Confocal microscope images were acquired as z-stacks having a *z*-interval of 15, 20, or 25 μm between two consecutive optical slices at a digital size of 512 × 512 pixels.

Detailed morphological analyses were performed using a scanning electron microscope (SEM; Zeiss EVO LS15) and focused ion beam (FIB; Zeiss, Cobra, AURIGA 60). After fixation of cell cultures in 4% PFA overnight at 4 °C, cells were washed with HBSS three times, dehydrated in graded ethanol mixtures (50–100%), air-dried for 30 min at room temperature, and coated with gold (ScanCoat 6, Oxford). Prepared samples were captured using a SE1 detector at 10-kV filament tension.

### Immunofluorescence staining

EMS and healthy cells were first seeded onto coverslips (Zeiss, Oberkochen, Germany) and pre-treated with calystegines for 24 h. The remaining medium was after that removed; cells were washed with HBSS and fixed in 4% paraformaldehyde for 40 min at room temperature. Subsequently, the cells were permeabilized with 0.1% Triton X-100 for 15 min at room temperature, washed three times with HBSS, and incubated 45 min in blocking buffer containing 10% goat serum and 0.2% Tween-20 in HBSS. Primary antibodies against *LAMP2* (Abcam, Cambridge, UK) diluted with 1:500 in HBSS containing 1% goat serum and 0.2% Tween-20 were then applied to cells overnight at 4 °C. After washing of antibodies excess, cells were treated with goat anti-mouse secondary antibodies conjugated with atto-488 (1:1000, Abcam, Cambridge, UK) for 1 h in the dark, at room temperature in a humidified chamber. The immunostained cells were finally mounted in ProLong Gold Antifade containing DAPI (Life Technologies, Warsaw, Poland) and were visualized and photographed using a confocal microscope (Zeiss Cell Observer SD).

### Apoptosis analysis by flow cytometry

The percentage of EMS and healthy ASCs undergoing apoptosis after treatment with *H. albus* calystegines was assessed using the Muse Annexin V & Dead Cell Assay kit™ (Merck Millipore, Darmstadt, Germany) according to the manufacturer’s protocol. All treated and untreated cells were harvested, washed with HBSS, and stained with the Annexin V & Dead Cell Kit for 20 min at room temperature. The distribution of cells across the four populations: (i) non-apoptotic cells, not undergoing detectable apoptosis: Annexin V (−) and 7-AAD (−); (ii) early apoptotic cells, Annexin V (+) and 7-AAD (−); (iii) late apoptotic cells, Annexin V (+) and 7-AAD (+); and (iv) cells that have died through non-apoptotic pathway: Annexin V (−) and 7-AAD (+), was established using the Muse Cell Analyzer (Merck Millipore, Darmstadt, Germany).

### Changes in mitochondrial transmembrane potential determination

Changes in the mitochondrial membrane potential (MMP) were detected on the basis of a MitoPotential lipophilic cationic dye, combined to the 7-AAD dye as an indicator of cell death, using the Muse™ MitoPotential Assay kit (Merck Millipore, Darmstadt, Germany). After exposure to different concentrations of calystegines, i.e., 250 and 500 μg/ml or medium for control groups, EMS and healthy cells were washed with HBSS and stained with the fluorescent dyes for 30 min at 37 °C, and the percentage of depolarized cells (depolarized live + depolarized dead) was assessed by the mean of a Muse Cell Analyzer (Merck Millipore, Darmstadt, Germany).

### Intracellular reactive oxygen and nitrogen species analysis

Quantitative measurements of ROS and RNS, namely superoxide and nitric oxide radicals were performed by the flow cytometry-based analysis using the Muse® Oxidative Stress Kit and Muse® Nitric Oxide Kit (Merck Millipore, Darmstadt, Germany) according to the users’ guide instructions. Cells were first pre-treated with two different concentrations of calystegines (250 and 500 μg/ml), then washed with HBSS and incubated with the corresponding fluorescent dyes for 30 min at 37 °C. Determination of ROS^+^/NO^+^ versus ROS^−^/NO^−^ populations was achieved using a Muse Cell Analyzer (Merck Millipore, Darmstadt, Germany).

### Quantitative real-time reverse transcription polymerase chain reaction (qRT-PCR)

Total RNA contained within the calystegine-treated EMS cells as well as EMS and healthy untreated cells was collected using the TRIzol method according to the manufacturer’s instructions. RNA purity and concentration were measured using a nanospectrophotometer (WPA, Biowave II, Germany). Genomic DNA (gDNA) digestion and cDNA synthesis were performed using a RevertAid First Strand cDNA Synthesis Kit (Thermo Fisher Scientific, Warszawa, Poland) by the mean of a T100 Thermal Cycler (Bio-Rad, Hercules, CA, USA) following the manufacturer’s instructions.

Expression levels of targeted genes (Table 1) implicated in insulin signaling pathway (GLUT4, AKT, PI3K, IR, IRS, SREBP1C), adipogenesis (PPARγ, ADIPOQ, Lep), apoptosis (p53, Cas-9, BAX, Bcl-2, p21), ER stress (PERK, CHOP, BiP, eIF2-α), and autophagy (LC3, Beclin, Lamp2) were analyzed by real-time reverse transcription polymerase chain reaction (RT-PCR), using the SensiFAST SYBR Green Kit (Bioline, London, UK) in a CFX Connect™ Real-Time PCR Detection System (Bio-Rad). Reactions performed in a 10-μl volume were subjected to the following cycling conditions: 95 °C for 2 min, followed by 40 cycles at 95 °C for 15 s, annealing for 15 s, and elongation at 72 °C for 15 s. All results were normalized to glyceraldehyde 3-phosphate dehydrogenase (GAPDH) expression. The relative expression level was calculated by comparison of the tested groups with the control group using the 2^-ΔΔCQ^ method.

**Table 1:** Sequences of primers used in qPCR

Gene	Primer	Sequence 5′–3′	Amplicon length (bp)	Accession no.
GLUT4	F:	TGGGCTCTCTCCGTGGCCATCTT	658	NM_001081866.2
	R:	GCTGCTGGCTGAGCTGCAGCA		
Akt	F:	AAGGAGATCATGCAGCACCG	180	XM_014854427.1
	R:	CTCCATCGTGTCGTCTTGGT		
PI3K	F:	GACTTGCACTTGGGTGACATA	152	XM_014855332.1
	R:	TAAGTTCCCGGAAAGTCCCC		
IR	F:	CCGTTTGAGTCTGAGGGGTC	254	XM_014862015.1
	R:	ACCGTCACATTCCCGACATC		
IRS	F:	CTGCTGGGGGTTTGGAGAAT	254	XM_014862015.1
	R:	TAAATCCTCACTGGAGCGGC		
SREBP-1c	F:	TCAGCGAGGCGGCTTTGGAGCAG	80	XM_008542859.1
	R:	CATGTCTTCGATGTCGGTCAG		
PPARγ	F:	TCCCTGTTTGTGTACAGCCTT	191	XM_014846252.1
	R:	CTCCATGGCTGATTTCCCCT		
ADIPOQ	F:	GGAGATCCAGGTCTTGTTGG	162	XM_014843352.1
	R:	TCGGGTCTCCAATCCTACAC		
Lep	F:	CACACGCAGTCAGTCTCCTC	176	XM_014854289.1
	R:	CGGAGGTTCTCCAGGTCAT		
p53	F:	TACTCCCCTGCCCTCAACAA	252	U37120.1
	R:	AGGAATCAGGGCCTTGAGGA		
Cas-9	F:	CACCTTCCCAGGCTTTGTCT	224	XM_014836232.1
	R:	GGCTCTGGCCTCAGTAAGTT		
Bax	F:	TTCCGACGGCAACTTCAACT	204	XM_005596728.1
	R:	GGTGACCCAAAGTCGGAGAG		
Bcl-2	F:	TTCTTTGAGTTCGGTGGGGT	164	XM_014843802.1
	R:	GGGCCGTACAGTTCCACAA		
p21	F:	GAAGAGAAACCCCCAGCTCC	241	XM_003365840.2
	R:	TGACTGCATCAAACCCCACA		
PERK	F:	GTGACTGCAATGGACCAGGA	283	XM_014852775.1
	R:	TCACGTGCTCACGAGGATATT		
CHOP	F:	AGCCAAAATCAGAGCCGGAA	272	XM_014844003.1
	R:	GGGGTCAAGAGTGGTGAAGG		
BiP	F:	CTGTAGCGTATGGTGCTGCT	122	XM_023628864.1
	R:	CATGACACCTCCCACGGTTT		
eIF2-α	F:	AGTCTTCAGGCATTGGCTCC	489	XM_001488848.5
	R:	CCGAGTGGGACATGTATCGG		
LC3	F:	TTCTGAGACACAGTCGGAGC	128	XM_001493613.6
	R:	CTTTGTTCGAAGGTGTGGCG		
Beclin	F:	GATGCGTTATGCCCAGATGC	233	XM_014833759.1
	R:	AACGGCAGCTCCTCTGAAAT		
Lamp2	F:	GCACCCCTGGGAAGTTCTTA	147	XM_014831347.1
	R:	ATCCAGCGAACACTCTTGGG		
GAPDH	F:	GATGCCCCAATGTTTGTGA	250	NM_001163856.1
	R:	AAGCAGGGATGATGTTCTGG		

### Statistical analysis

All data are presented as the mean ± standard deviation (SD) of at least three independent experiments. Statistical evaluation was realized using GraphPad Prism 5.0 (San Diego, CA, USA). Levels of significance were calculated by using one-way analysis of variance (ANOVA) with Dunnett’s post hoc multiple comparison test. Asterisk (*) and hash (#) sign indicated statistical significance in EMS_ASCs_ control or *calystegine*-treated groups versus healthy control or EMS-treated control versus *calystegine*-treated groups, respectively. *p* values less than 0.05 (*p < 0.05*) were annotated with one asterisk/hash (*/#), *p* < 0.01 with two asterisks/hashes (**/##), and *p* < 0.001 with three asterisks/hashes (***/###).

## Results

### Calystegine identification

**Fig. 1:**
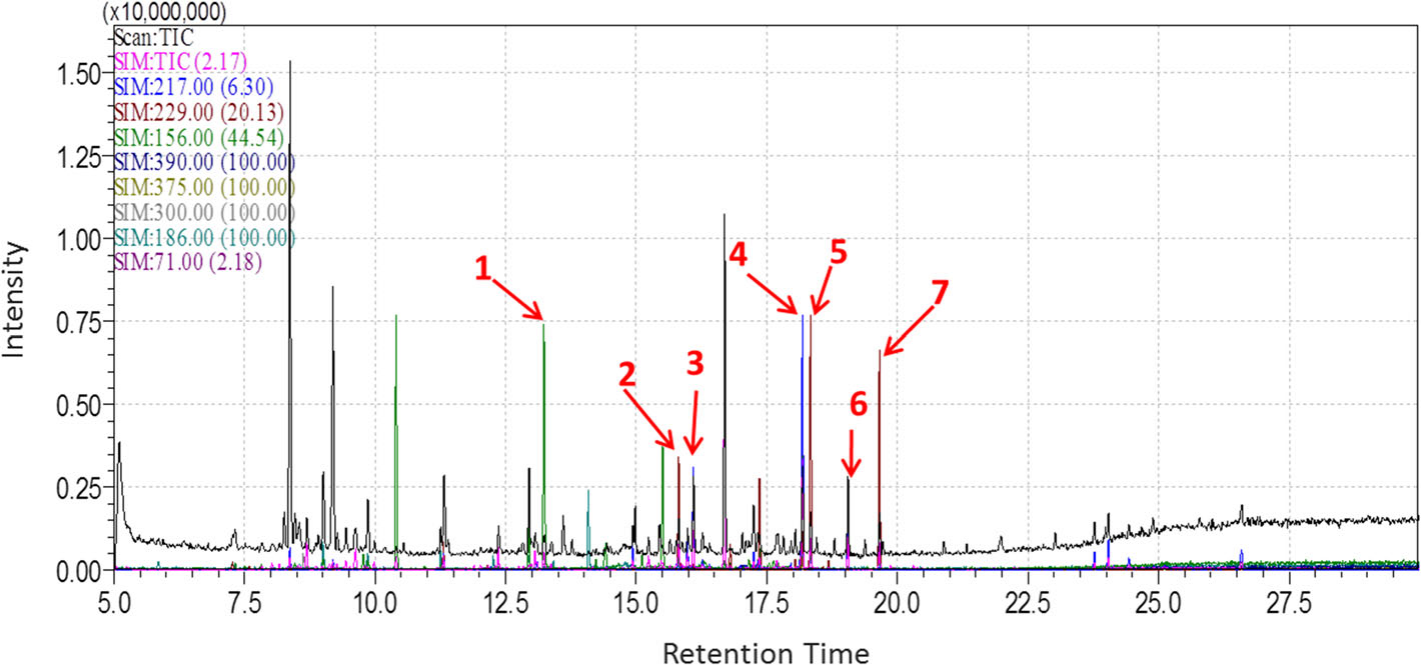
GC-MS analysis of *Hyoscyamus albus* seed extract. 217 *m*/*z*: calystegines B; 229 *m*/*z*: calystegine B2; 156 *m*/*z*: calystegines A; 390 *m*/*z*: calystegine N1; 375 *m*/*z*: calystegine C1; 189 *m*/*z*: calystegine B1; 71 *m*/*z*: octadecane; 1: calystegine A5; 2: calystégine A3; 3: calystegineA5 Gly.; 4: calystegine B4; 5: calystegine B1; 6: calystegine N1; 7: calystegine B2 [24]

Results from the GC-MS characterization of the calystegine-rich extract of *Hyoscyamus albus* seeds are shown in Table 1. Identification made from retention times and fragmentation patterns (Fig. 1) specific to each compound has demonstrated the presence of all the B group calystegines (B1, B2, and B4) with predominance of calystegine B4; in addition, three calystegines from the A group (A3, A5, and A5 glycoside) as well as calystegine N1 were also evidenced in the extract [24].

### Total calystegines improved cell survival through stimulation of cell proliferation and reduced apoptosis in EMS_ASCs_

**Fig. 2:**
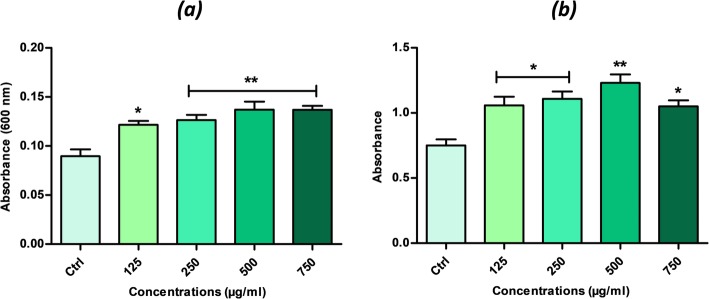
Dose response effects of *Hyoscyamus albus* calystegines on EMS_ASCs_ cell viability and proliferation. **a** Histograms represent the average absorbance at 600 nm of the metabolized rezasurin dye by treated and untreated living cells. **b** Bar charts represent the mean of the absorbance of the BrdU level incorporated in the neosynthesized DNA of the treated and untreated cells. The results are expressed as the mean of 3 different experiments ± SD. Asterisk (*) refers to the comparison of treated groups to untreated EMS cells. *p < 0.05, **p < 0.01, ***p < 0.001

To test the effect of total calystegines on the viability of EMS_ASCs_ cells, the cells were treated with the indicated concentrations of calystegines for 72 h. After co-culture, their viability was investigated by rezasurine-based assay. Calystegines induced a remarkable increase in metabolically active living cells after treatment (Fig. 2a), although they were significant over the concentration of 125 μg/ml (*p* < 0.05) and appeared to be more effective in a range of concentrations from 250 to 750 μg/ml (*p* < 0.01) after 72-h exposure to cells as compared to untreated EMS cells. In fact, calystegines enhanced EMS ASC survival by almost 32% at the lowest concentration (125 μg/ml); thereafter, the survival rate has risen to 48% at 500 μg/ml with no significant difference between the three highest concentrations, indicating that these molecules exert their effect in a dose-independent manner (Table 2).

**Table 2:** Calystegines’ composition in *Hyoscyamus albus* seed extract [23]

Compound number	Calystegines	Retention time (min)	Concentration (μg/g DW)
1	Cal A_5_	13.2	45.91 ± 9.24
2	Cal A_3_	15.8	54.90 ± 3.95
3	Cal A_5_ glycoside	16.1	197.76 ± 21.96
4	Cal B_4_	18.12	212.54 ± 8.12
5	Cal B_1_	18.34	91.25 ± 5.71
6	Cal N_1_	19.05	180.22 ± 10.9
7	Cal B_2_	19.70	87.14 ± 6.35

In order to determine whether calystegines extracted from *H. albus* promote not only cell survival but also cell proliferation, BrdU assay which detects cell division by measuring new DNA synthesis was performed after cell exposure to various concentrations of polyhydroxylated alkaloids. As shown in Fig. 2b, under calystegine treatment, BrdU incorporation significantly increased for all tested concentrations in comparison to untreated cells (*p* < 0.05; *p* < 0.001); however, results indicated also that pro-proliferative effect of calystegines was not dependent on concentration. In the presence of 500 μg/ml of alkaloids, BrdU incorporation increased by almost 47% compared to control levels (*p* < 0.01). Other concentrations nevertheless have modulated BrdU incorporation from 30 to 36% compared to untreated EMS phenotype (*p* < 0.05).

### Cell morphology

**Fig. 3:**
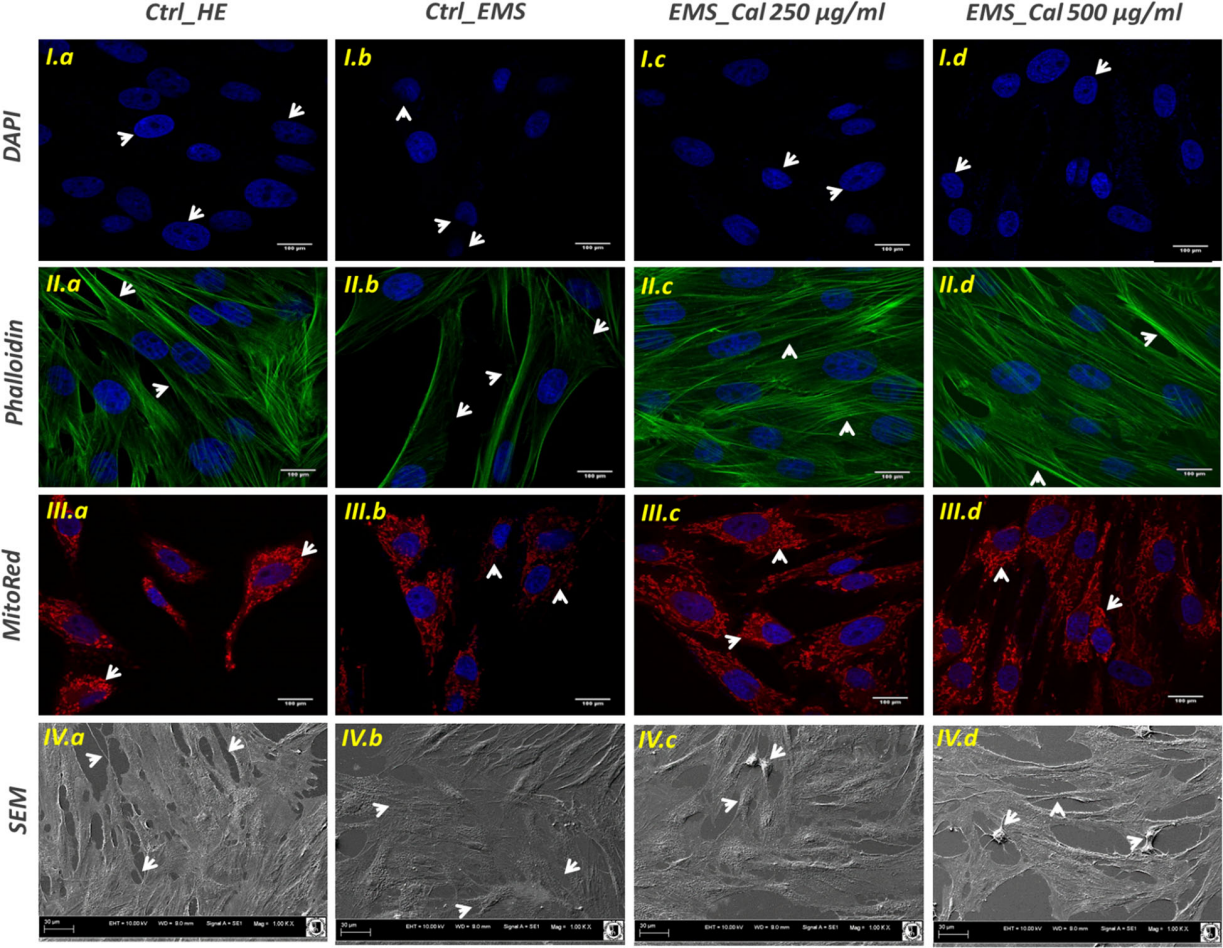
Analysis of general architecture of healthy and EMS untreated and treated ASC cells. DAPI-, MitoRed-, and Phalloidin-labeled cells were observed using an inverted epi-fluorescent confocal microscope; scale bar size 100 μm; magnification was set at 60-fold. Cell surface and shape were assessed by the mean of scanning electron microscope; scale bar size 30 μm; images were acquired under 1000-fold magnification. *a* Healthy control cells; *b* EMS control cells; *c* EMS cells treated with 250 μg/ml of calystegines; *d* EMS cells treated with 500 μg/ml of calystegines

SEM micrographs of EqASC healthy phenotype (Fig. 3) showed that cells adhered and spread on the surface of their substrate. Moreover, healthy cultures displayed fibroblast-like and well elongated morphology and developed long lamellipodia and filopodia structures for adjacent cell connection (micrograph *IV.a*). EMS_ASCs_ cells exhibit a slightly altered shrank and cracked surface; their surface appeared flat and microvilli did not extend from the cell surface. Relief of cell surface seemed to be somewhat inconspicuous (micrograph *IV.b*). Treatment of EMS cells with calystegines did not induce any extensive and obvious alteration of the general structure of the cells. Cell confluence was more pronounced after alkaloid treatment, the cell surfaces appeared embossed, and some cells showed bulging relief. Numerous microvilli started to extend from the cell surfaces (micrographs *IV.c and IV.d*). Confocal microscopy revealed a larger number of oval nuclei in the calystegine-treated EMS cells compared to untreated EMS cells that showed a number of strands of DAPI-stained nuclei, some of which were irregularly shaped; moreover, one or more prominent nucleoli were observed in the nucleus of both untreated and treated cells (micrographs *I.b*, *A.c*, and *I.d*). In the same way, cells labeled with MitoRed showed more abundant fluorescent red spots in the cytoplasm of treated cells corresponding to the active mitochondria (micrographs *III.c and III.d*), compared to the smaller mitochondrial regions of EMS control cells (micrographs *III.b*). Finally, the analysis evidenced a relatively well-structured and dense β-actin network (micrographs *II.c and II.d*), comparable to that of the healthy cells; actin filaments appeared fairly regular and uniformly distributed (Fig. 3).

### Effect of calystegines in EMS ASC apoptosis

**Fig. 4:**
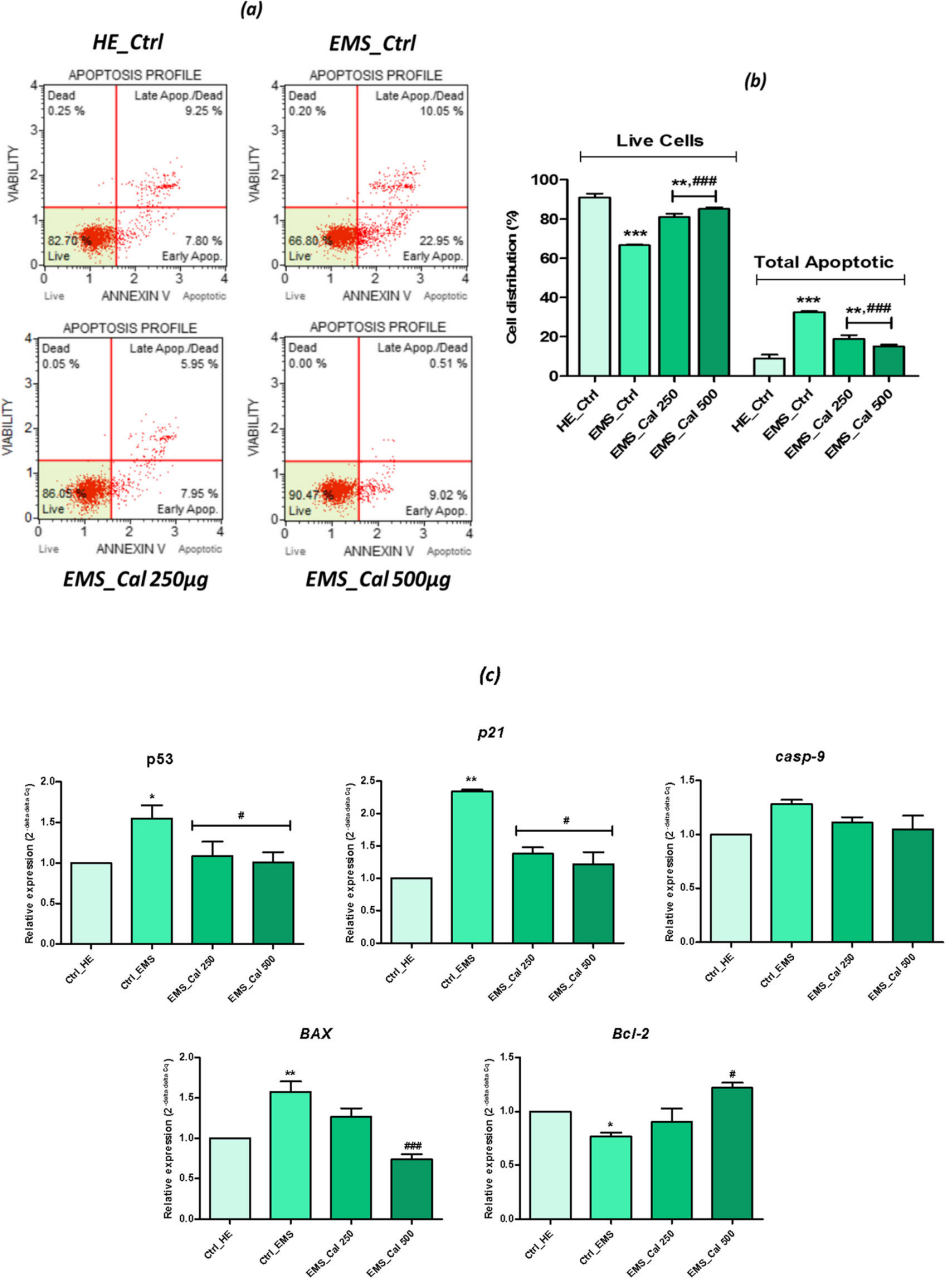
Anti-apoptotic effects of calystegines on EMS_ASCs_ cells. **a** Flow cytometry-based Annexin V/7-AAD representative plots. **b** Bar chart representation of total living and total apoptotic cell distribution expressed in percentage. **c** RT-qPCR analysis of relative expression of the main related apoptotic genes. *Ctrl_HE* healthy control cells, *Ctrl_EMS* EMS control cells, *EMS_Cal 250* EMS cells treated with 250 μg/ml of calystegines, *EMS_Cal 500* EMS cells treated with 500 μg/ml of calystegines. Values are mean ± SD (*n* = 3). Asterisk (*) refers to the comparison of EMS treated and untreated groups to untreated healthy cells, Hashtag (#) refers to the comparison of EMS treated groups to EMS untreated cells. *^/#^*p* < 0.05, **^/##^*p* < 0.01, ***^/###^*p* < 0.001

The effects of total calystegines extracted from *H. albus* seeds on apoptosis were evaluated by using Annexin V/7-Aminoactinomycin D (7-AAD) staining through flow cytometry. Translocation of phosphatidylserine (PS) to the outer leaflet of the cellular membrane is the key step in the early stages of apoptosis. Annexin V selectively binds to PS and helps to identify cells undergoing apoptosis. 7-AAD is a red fluorescent, live-cell impairment chemical compound that intercalates in double-stranded DNA, with a high affinity for GC-rich regions [26]. As showed in Fig. 4b, EMS_ASCs_ cells were characterized by an increased apoptotic tendency and significantly more pronounced by a total apoptotic cell count of 32.56 ± 0.46% compared to only 8.96 ± 0.84% of apoptotic cells for the healthy control cells (*p* < 0.001). Flow cytometric analysis demonstrated that incubation of EMS cells in the presence of calystegines has remarkably reduced the number of total apoptotic cells *(p* < 0.001) by nearly 1.7-fold for the concentration of 250 μg/ml and up to 2.7-fold at the highest concentration, i.e., 500 μg/ml.

To investigate the possible association between calystegine-reduced EMS_ASCs_ apoptosis and changes in gene expression of main related markers involved in apoptotic pathway regulation, the expression levels of the anti-apoptotic and pro-apoptotic genes were examined after extract treatments. Figure 4c shows that EMS_ASCs_ cells exhibit significant higher induction of main pro-apoptotic genes, namely *p53*, *p21*, and *Bax* when compared to normal untreated ASCs (*p* < 0.05; *p* < 0.001). Moreover, the level of *Bcl-2* mRNA appeared to be drastically reduced in EMS cells as compared to healthy phenotype (*p* < 0.05). RT-qPCR results (Fig. 4c) confirmed the anti-apoptotic effect of calystegines on EMS cells; indeed, supplementation of EMS cell cultures by the two concentrations of alkaloids (250 and 500 μg/ml) made it possible to significantly reduce expression of pro-apoptotic *p53* and *p21* genes, suggesting a possible interference with this activating apoptosis pathway (*p* < 0.05). Even more interesting, calystegines have exerted a regulating effect on the *Bcl-2/Bax* signaling pathway; thus, at the highest concentration of 500 μg/ml of calystegines, the results demonstrated that these alkaloids promoted EMS cell survival by modulating the expression of the *Bcl-2*-survival gene and downregulating the expression of *Bax* gene (*p* < 0.001).

### Effect of calystegines on ROS and NO accumulation

**Fig. 5:**
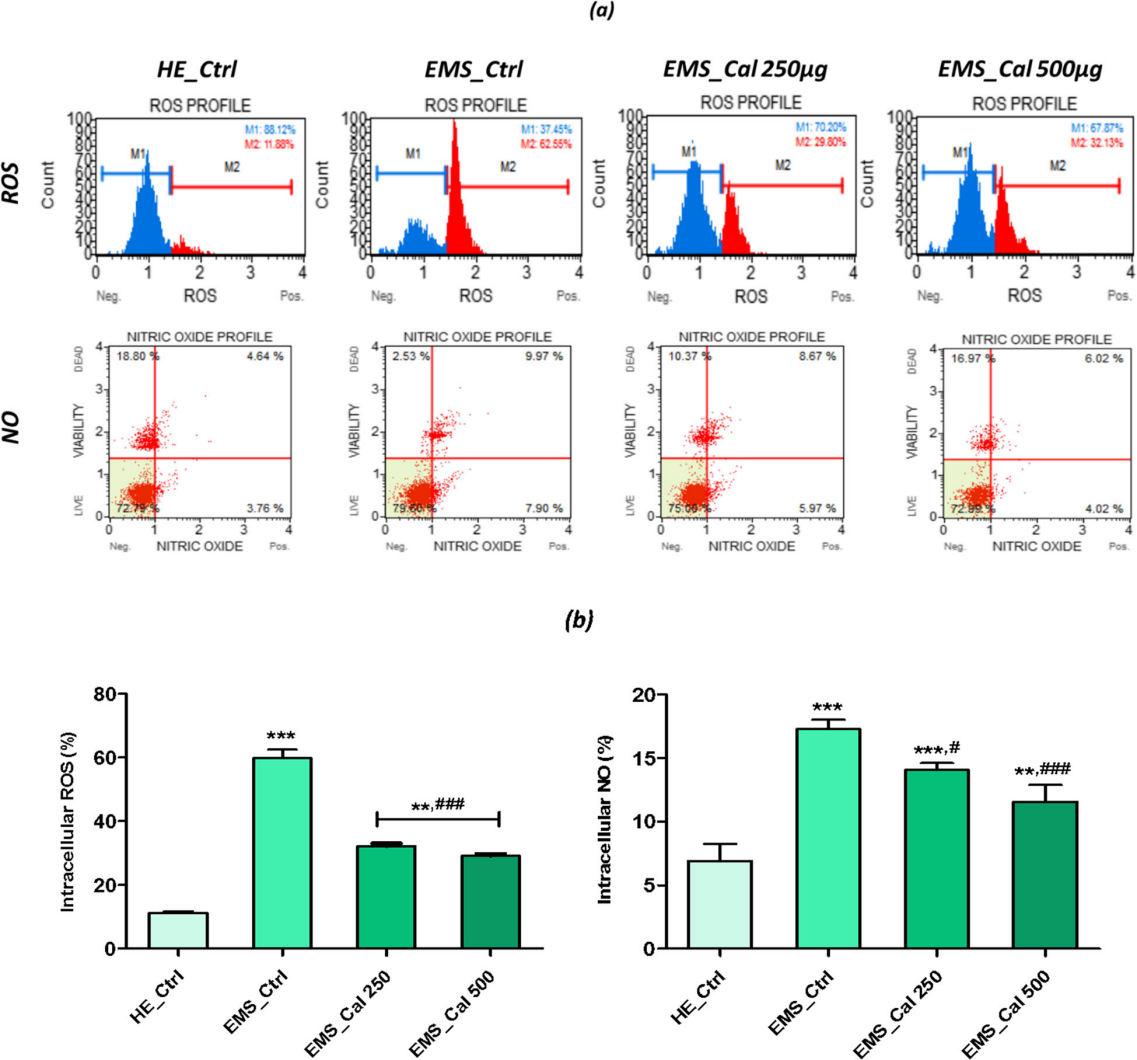
Effect of calystegines on ROS and NO levels in EMS_ASCs_ cells. **a** Representative plots depicting cell stained with DHE and DAX-J2 Orange dyes and evaluated using a flow cytometer. **b** Each bar represents the mean ± SEM of three independent experiments for intracellular ROS and NO. *Ctrl_HE* healthy control cells, *Ctrl_EMS* EMS control cells, *EMS_Cal 250* EMS cells treated with 250 μg/ml of calystegines, *EMS_Cal 500* EMS cells treated with 500 μg/ml of calystegines. Asterisk (*) refers to the comparison of EMS treated and untreated groups to untreated healthy cells. Hashtag (#) refers to comparison of EMS treated groups to EMS untreated cells. *^/#^*p* < 0.05, **^/##^*p* < 0.01, ***^/###^*p* < 0.001

The ability of the *Hyoscyamus albus* calystegines to reduce reactive oxygen species (ROS) as well as nitric oxides (NOs) was evaluated using the cell permeable dyes dihydroethidium (DHE) and DAX-J2 Orange respectively. ROS and NO levels (Fig. 5b) were greatly increased in EMS_ASCs_ cells, to about 48.62% and 10.35% of control ROS and NO values respectively (*p* < 0.001). As shown in Fig. 5a and b, the polyhydroxylated alkaloids exhibited a high antioxidant potential, by substantially reducing the intracellular levels of ROS and NO produced by EMS cells (*p* < 0.001). In fact, at a concentration of 500 μg/ml, the percentage of ROS and NO decreased by nearly 48.67% and 66.87% respectively compared to the EMS control cells.

### Effect of calystegines on mitochondrial membrane potential

**Fig. 6:**
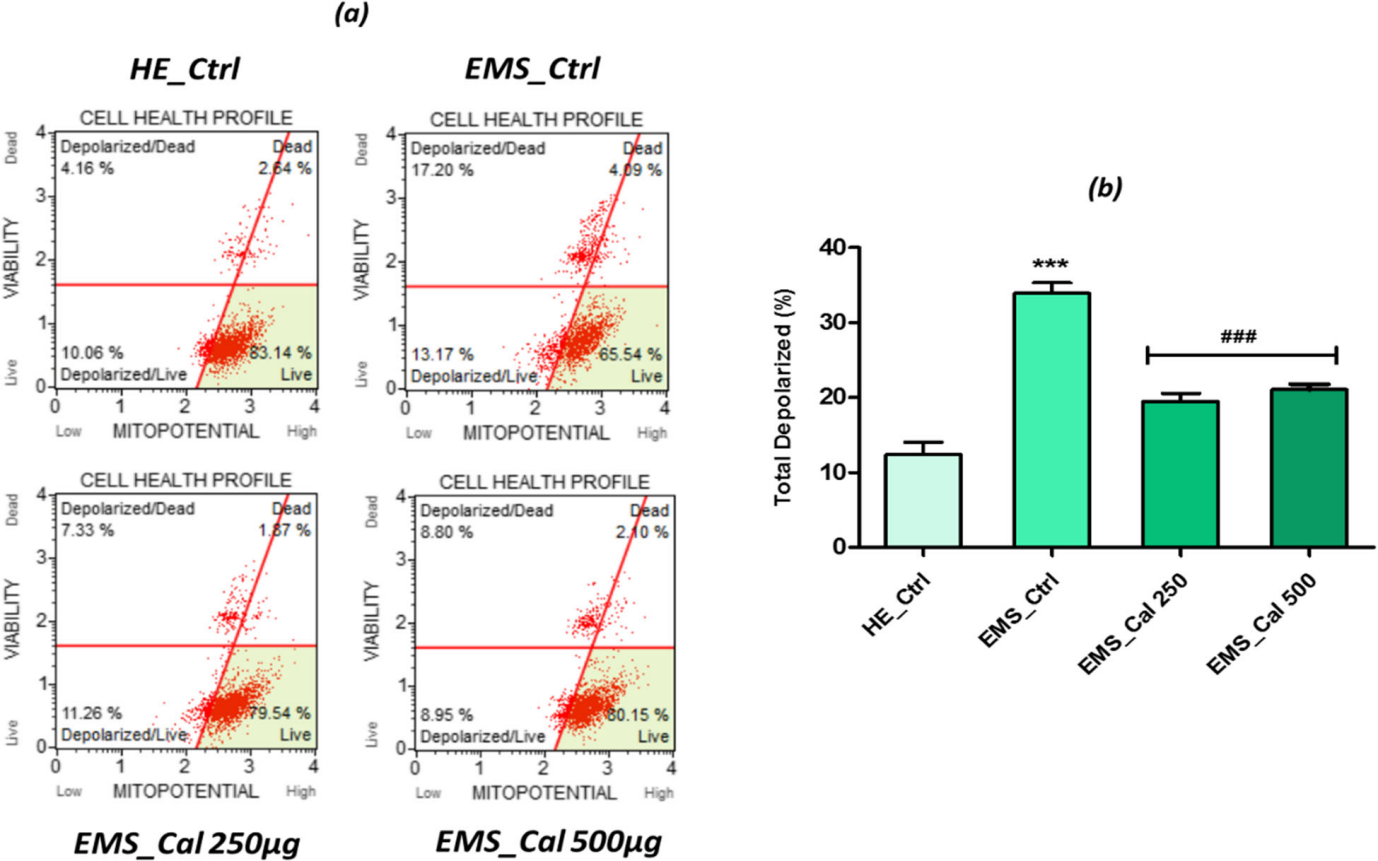
Mitochondrial membrane potential analysis. **a** Scattered blot representation of live and dead depolarized cell percentages for one representative experiment. **b** Histograms represent the average percentages ± SD of total depolarization for three repetitions. *Ctrl_HE* healthy control cells, *Ctrl_EMS* EMS control cells, *EMS_Cal 250* EMS cells treated with 250 μg/ml of calystegines, *EMS_Cal 500* EMS cells treated with 500 μg/ml of calystegines. Asterisk (*) refers to the comparison of EMS treated and untreated groups to untreated healthy cells. Hashtag (#) refers to the comparison of EMS treated groups to EMS untreated cells. *^/#^*p* < 0.05, **^/##^*p* < 0.01, ***^/###^*p* < 0.001

The mitochondrial membrane potential (*ΔΨm*) is a reliable indicator of mitochondrial-dependent apoptosis which can be quantified by applying fluorescent staining [27]. Functional status of mitochondria in EMS untreated cell analysis (Fig. 6) showed a dramatically decreased mitochondrial membrane potential as compared to healthy ASCs (*p* < 0.001), which is in concordance to results obtained in apoptosis assay. EMS cells exhibited a mean of 33.91 ± 1.06% of total depolarized cells, when normal ASCs showed only 12.41 ± 1.03% for the same population. Therapeutic treatment with calystegines at both tested concentrations resulted in a near-complete recovery of MMP, as no significant differences from healthy cells were recorded (Fig. 6). In fact, treated EMS cells presented a significantly greater MMP (*p* < 0.001) than untreated cells.

### Effect of calystegines on gene expression of insulin resistance markers

**Fig. 7:**
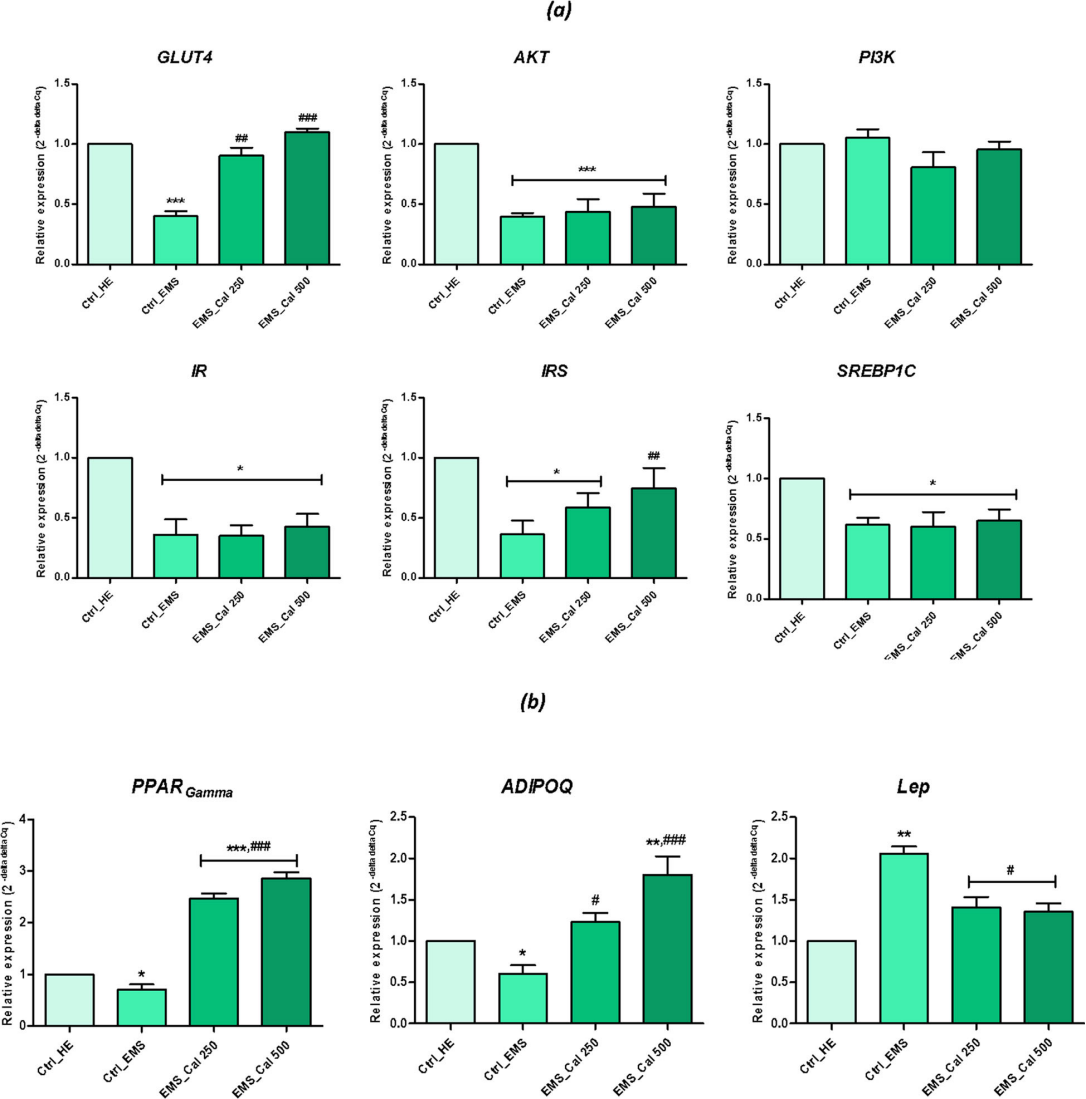
RT-PCR analysis of insulin resistance markers after calystegine treatment on EMS_ASCs_ cells. **a** Gene expression levels of key factors involved in insulin signaling pathway. **b** Gene expression levels of lipid metabolism markers. *Ctrl_HE* healthy control cells. *Ctrl_EMS* EMS control cells, *EMS_Cal 250* EMS cells treated with 250 μg/ml of calystegines, *EMS_Cal 500* EMS cells treated with 500 μg/ml of calystegines. Results are expressed as mean of three independent experiments ± SD. Asterisk (*) refers to the comparison of EMS treated and untreated groups to untreated healthy cells. Hashtag (#) refers to the comparison of EMS treated groups to EMS untreated cells. *^/#^*p* < 0.05, **^/##^*p* < 0.01, ***^/###^*p* < 0.001

In order to assess the possible effect of total calystegines on insulin resistance, which is characteristic in EMS, relative expression of key factors involved in the insulin signaling pathway was evaluated (Fig. 7). mRNA analysis of the EMS control group resulted in a significant decrease in main related factors implicated in insulin signaling pathway (Fig. 7a); indeed, expression of GLUT4, insulin receptor (IR), insulin receptor substrate (IRS), *AKT*, and *SREBP1C* were significantly downregulated under metabolic syndrome condition (*p* < 0.05; *p* < 0.001). The effect of calystegines on the resistance of EMS cells to insulin was found to be selective and limited to the upregulation of the gene encoding the *GLUT4* glucose receptor as well as the IRS gene only; expression of the rest of targeted genes in this study was found to be unchanged under calystegine action. Iminosugars of *H. albus* thus modulated the expression of *GLUT4* receptor mRNA by 2.7-fold compared to that of untreated EMS cells (*p* < 0.001), as well as for the expression of *IRS* gene, which proved to be upregulated by closely 2.2-fold (*p < 0.01*), suggesting that these molecules would be endowed with an insulin mimetic effect.

To explore the molecular mechanisms by which, calystegines improved lipid metabolism in EMS_ASCs_ cells, expression of adiponectin, leptin, and PPAR-γ were examined (Fig. 7b). Adiponectin is an insulin sensitizing protein secreted from adipose tissue to increase peripheral glucose utilization in the liver and muscle. Adiponectin expression was strongly decreased in EMS ASC control cells and significantly increased in calystegine-treated cells (*p* < 0.001). Leptin expression analysis resulted in higher levels of *Lep* gene expression in EMS cells compared to healthy cells (*p* < 0.01); total calystegine supplementation to EMS cells markedly decreased leptin expression by 35% at the highest concentration (*p* < 0.05). Peroxisome proliferator-activated receptor gamma (PPAR-γ)-related nuclear hormone factor is known to manly enhance the expression of a number of genes encoding proteins involved in glucose and lipid metabolism; RT-qPCR examination of EMS_ASCs_ cell mRNA indicated a remarkable decrease in the level of *PPAR-γ* gene expression as compared to healthy cells (*p* < 0.05). However, exposure of EMS cells to both doses of calystegines led not only to a restoration of gene expression (*p* < 0.001) but also to its upregulation when compared to healthy cells (*p* < 0.001), which would suggest a stimulating effect on the lipid metabolism signaling pathways.

### Effect of calystegines on ER stress markers

**Fig. 8:**
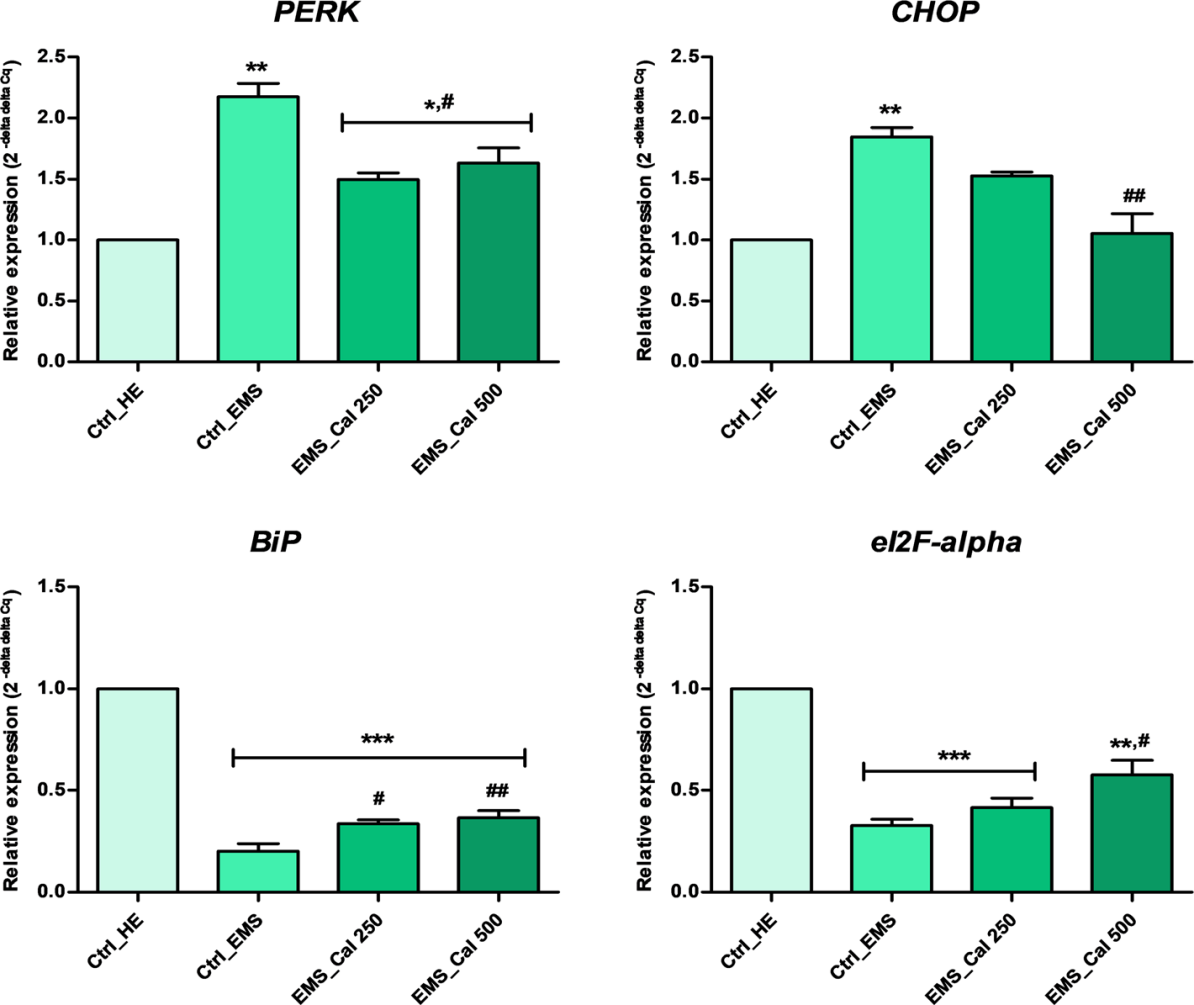
RT-PCR analysis of main ER stress-related markers after calystegine treatment on EMS_ASCs_ cells. Data are expressed as mean of three independent experiments ± SD. *Ctrl_HE* healthy control cells, *Ctrl_EMS* EMS control cells, *EMS_Cal 250* EMS cells treated with 250 μg/ml of calystegines, *EMS_Cal 500* EMS cells treated with 500 μg/ml of calystegines. Asterisk (*) refers to the comparison of EMS treated and untreated groups to untreated healthy cells. Hashtag (#) refers to the comparison of EMS treated groups to EMS untreated cells. *^/#^*p* < 0.05, **^/##^*p* < 0.01, ***^/###^*p* < 0.001

To evaluate the extent of ER dysfunction in EMS_ASCs_ cells following calystegine exposure, expression of several ER stress markers was measured 24 h after treatment with alkaloids. As summarized in Fig. 8, the levels of *PERK* and *CHOP* were high in the EMS group as opposed to the healthy group (*p* < 0.01), whereas post-treatment with calystegines downregulated significantly these ER stress marker expression (*p* < 0.05; *p* < 0.01) reaching 25.8% and 42.97% decrease for both *PERK* and *CHOP* respectively at the highest concentration (i.e., 500 μg/ml). By contrast, both *BiP* and *eIF2-α* transcripts were 5-fold and 3-fold downregulated respectively in EMS ASCs comparatively to the non-stressed cells (*p* < 0.001). Supplementation of EMS cell cultures with calystegines resulted in a marked upregulation of *BiP* and *eI2F-α* expression (Fig. 8) when compared with the no treatment EMS control (*p* < 0.05; *p* < 0.01). The extent of gene expression restoration exerted by calystegines thus reached 43.22% for *BiP* and around 45.12% for *eIF2-α* at 500 μg alkaloids.

### Effect of calystegines on autophagy

**Fig. 9:**
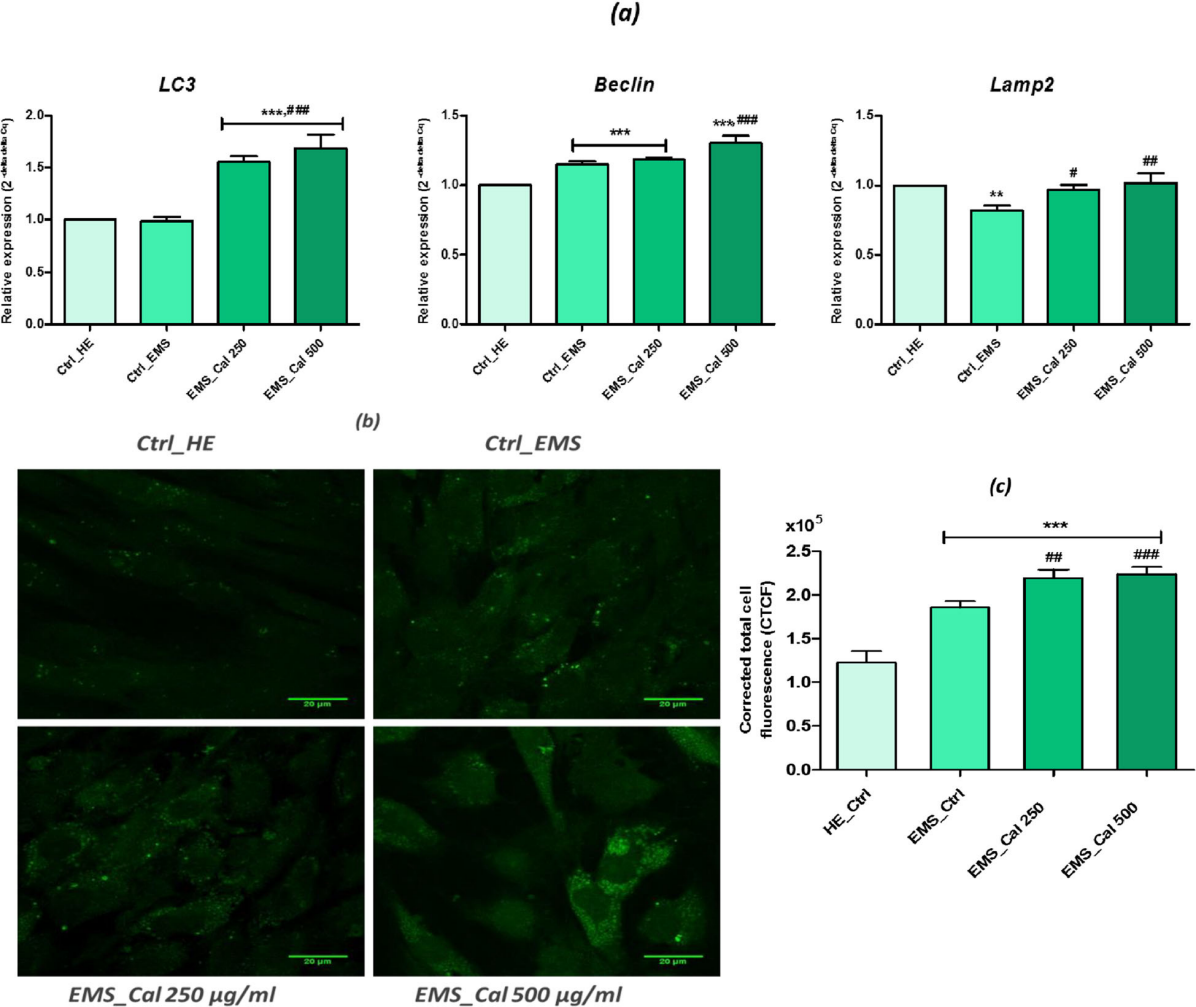
Assessment of autophagy in EMS_ASCs_ treated with calystegines. **a** qRT-PCR analysis of key markers involved in autophagy process. **b** Representative photomicrographs of Lamp2 immunofluorescent staining acquired using a confocal microscope; scale bar size 20 μm; magnification was set at 60-fold. **c** Bar chart representation of corrected total cell fluorescence (CTCF) for Lamp2 calculated using the ImageJ software. Values are expressed as mean of three independent experiments ± SD. *Ctrl_HE* healthy control cells, *Ctrl_EMS* EMS control cells, *EMS_Cal 250* EMS cells treated with 250 μg/ml of calystegines, *EMS_Cal 500* EMS cells treated with 500 μg/ml of calystegines. Asterisk (*) refers to the comparison of EMS treated and untreated groups to untreated healthy cells. Hashtag (#) refers to the comparison of EMS treated groups to EMS untreated cells. *^/#^*p* < 0.05, **^/##^*p* < 0.01, ***^/###^*p* < 0.001

Autophagic capacity of EMS_ASCs_ cells was monitored after calystegine treatment by *LC3*, *Beclin*, and *Lamp2* evaluation using qRT-PCR (Fig. 9a). The outcomes of gene transcript analysis showed that expression of *LC3*, *Beclin*, and *Lamp2* was substantially increased (*p* < 0.001) under the effect of the polyhydroxylated alkaloids which presumably modulated the autophagic signaling pathways. The relative expression of the gene encoding *LC3* was therefore stimulated by nearly 41.21% compared to that of untreated EMS cells and about 40.61% compared to the basal expression of the healthy cell group. As for *Beclin*, the transcription rate reached 12% more than that observed in the untreated cells and culminated 23.3% of overexpression in respect to the healthy control. Additionally, calystegines were able to modulate nearly 19.40% of *Lamp2* gene expression when compared to untreated EMS cells (Fig. 9a). Immunofluorescence analysis of lamp2 in EMS_ASCs_ cells revealed a vesicular distribution patterns for lamp2 in all treated and untreated groups (Fig. 9b); however, stronger immunoreactivity in EMS cells was monitored compared to healthy cells indicating a stronger activation of the autophagy process in diseased cells. Indeed, the analysis of the corrected total cell fluorescence (CTCF) showed a 1.5-fold fluorescent signal intensity (Fig. 9c) greater for EMS cells (*p* < 0.001). The 24-h treatment with calystegines permitted to induce a significant lamp2 accumulation within the cells, which appeared to be stronger than that in both EMS and healthy cells (*p* < 0.001). The immunological staining results thus showed 1.2-fold and 1.83-fold higher relative fluorescent signal for cells having received 500 μg/ml calystegines as compared to EMS and healthy groups respectively, involving a consequent modulation of autophagy.

## Discussion

In this study, we explored the recovery effects of total calystegines extracted from *Hyoscyamus albus* seeds, on alterations induced during metabolic syndrome in equine adipose-derived stromal cells. The equine metabolic syndrome is an endocrinopathy affecting horses and ponies, which is characterized by a conflation of three main conditions, namely insulin dysregulation, abnormal adipose distribution, and a high risk for laminitis, thus making a number of physiological and cellular disturbances and alterations [28].

Results from biocompatibility testing showed that calystegines do not exert any cytotoxicity on EMS ASCs up to a maximum of 700 μg/ml; moreover, application of calystegines allowed to reverse the trend of EMS cell apoptosis at least partially and modulated their proliferation, evidenced by the high incorporation rate of BrdU into the neosynthesized DNA. Several previous studies have already clearly demonstrated that ASCs isolated from equine subjects suffering from metabolic syndrome were strongly characterized by a decrease in their proliferative potential rate and were in part highly prone to apoptosis and senescence in comparison to healthy cell phenotype [13, 16, 17, 29, 30]. The monitoring of the gene’s expression involved in apoptosis pathway has shown a remarkable overexpression of *p53*, *p21*, and *Bax* genes, which largely trigger cell death in EMS_ASCs_; Marycz et al. [31] have highlighted the high abundance of *p53*, *p21*, and *Bax* and *Casp9* transcripts for the same cell type. The same study showed significant impairment in the anti-apoptotic *Bcl2* gene expression. The ability of calystegines to promote cell viability was closely related to a significant repression of the apoptosis genes’ transcription (*p53*, *p21*, *Bax*) and, more interesting, to the restoration and upregulation of the cell survival gene *Bcl2*. Apoptosis is known to play a pivotal role in the pathogenesis processes of various diseases, including metabolic syndrome [32]. Bcl-2 protein is a key factor in the regulation and inhibition of apoptosis, and its overexpression can effectively prevent apoptosis [33]. It has been demonstrated that the promoter region of the *Bcl-2* gene comprises a highly cAMP-responsive element site (CRE) and its expression is upregulated through the CREB transcription factor [34]. Thus, anti-apoptotic effect of calystegines, manifested by the upregulation of the *Bcl-2* gene, could be partly related to modulation of cAMP production. Several studies have shown that certain secondary metabolites, such as hyoscyamine and atropine, which are like calystegines tropane metabolism-related alkaloid, possess the capacity to stimulate the cAMP pathway mainly by enhancing its synthesis [35–37].

To further investigate the context in which EMS_ASCs_ cells were prone to increased apoptosis, mitochondria’s membrane dynamics was analyzed. EMS_ASCs_ cells were strongly characterized by decreased MMP compared to normal cells, and subsequent treatment with calystegines allowed a restoration of the mitochondrial membrane integrity. One of the many key pathophysiological pathways involved in the development of metabolic syndrome is mitochondrial dysfunction, which is known to be triggered by genetic factors in both mitochondrial and nuclear genomes as well as many other risk factors such as IR, hyperglycemia, aging, and hypoxia [38]. These mitochondrial dynamics alterations are typically characterized by impaired beta-oxidation, decreased mitochondrial membrane potential (MMP), excessive generation of ROS, and overall reduced energy metabolism [39]. As one of the most prominent manifestations of altered mitochondria, apoptosis occurs when changes in the inner mitochondrial membrane results in an opening of the mitochondrial permeability transition (MPT) pore, loss of the mitochondrial transmembrane potential, and release of cytochrome c, caspases activators and *Smac/DIABLO* proteins [40, 41]. Fagomine and deoxynojirimycin (DNJ) are similar to calystegines, two polyhydroxylated alkaloids that have already shown the ability to restore MMP following a collapse induced by hyperglycemia through activation of AMPK, which is a regulator of cellular energy, and whose activity is closely linked to mitochondrial function [42]. It is therefore reasonable to postulate that beneficial effect of calystegines on mitochondrial function may follow the same molecular mechanism of action and that this may also be another pathway in the suppression of apoptosis. Overproduction of ROS during metabolic dysfunctions usually results in cellular damage that may be irreversible. The metabolic syndrome condition is characterized by a fairly high degree of oxidative damage that is usually manifest by a dramatic collapse of cellular antioxidant defenses, with mainly a decrease in the activity of superoxide dismutase and catalase and a concomitant increase in malondialdehyde levels, lipid peroxidation degree, protein carbonylation, and xanthine oxidase activity [43]. Following treatment of EMS_ASCs_ cells with calystegines, levels of produced ROS and NO were significantly reduced compared to untreated cells. During metabolic syndrome, increased levels of glucose lead to overproduction of ROS which enhances mitochondrial defect and antioxidant capacity impairment [44]. Additionally, ROS produced in the mitochondria during OXPHOS process can trigger mitochondrial dysfunction by interacting with mitochondrial and cellular components such as DNA, proteins, lipids, and other molecules [45, 46]. Further accumulation of mtDNA mutations within mitochondria in turn could damage its structure and functions and consequently alters antioxidant defense systems [47]. Previous study has already reported on the potent in vitro antioxidant effects of calystegines. Indeed, these alkaloids are endowed with strong antiradical effect and reducing power toward various free radicals such as DPPH, ABTS, and AAPH. This potential has been attributed to the high degree of hydroxylated substitutions and was related to their several OH-moieties [24]. Moreover, it was demonstrated that increasing the degree of hydroxylation of the nortropane ring results in the enhancement of their biological activities [48].

Insulin resistance (IR) together with obesity is considered to be one of the main causes underlying the onset of metabolic syndrome. Many tissues, including skeletal muscle, liver, and adipose tissue, may then be affected by this resistance [49]. The present investigation demonstrated that signaling pathways of insulin and lipid metabolism were substantially altered in EMS_ASCs_ cells; a collapse of the genes’ expression of key markers involved in glucose (*GLUT4*, *IR*, *IRS* ...) and lipid (*ADIPOQ*, *PPAR-γ*) metabolism has thus been observed. Overproduction of lipids and changes in substrate metabolism represent the basis of chronic tissue inflammation, which contributes greatly to the development of peripheral insulin resistance. Adipose tissue accumulation of bioactive lipid species activates pro-inflammatory signaling pathways and proteins kinases C (PKCs) which are known to be involved in the disruption of insulin signal transduction by altering key phosphorylation events and key protein-to-protein interactions [50]. Impairment of insulin receptivity at the IRS level results from a stress of kinase activation (c-Jun N-terminal kinase (JNK) and nuclear factor-κB (IκB) kinase (IKK)β) concomitantly with a defect in IRS phosphorylation. This results in a marked decrease in PI3K activity, thus disabling downstream signal transduction, which implies mainly the serine/threonine kinase Akt and consequently inhibits insulin-stimulated GLUT4 translocation to the plasma membrane [48, 51]. Adipose tissue strongly participates in insulin resistance. Overproduction of leptin and reduction of adiponectin were thus previously identified during obesity and are recognized to be partly involved in insulin resistance. When adiponectin binds to its receptors, AMP-activated protein kinase (AMPK) as well as receptors activated by peroxisome proliferators (PPAR) phosphorylation occurs and triggers an increase in fatty acid oxidation and glucose uptake [52]. PPARs are thus responsible for modulating the expression of genes involved in lipid metabolism, and their activation promotes lipid oxidation and lipogenesis, stimulates adipocyte differentiation, and increases insulin sensitivity in mature adipocytes [53]. Leptin, for its part, acts directly on the hypothalamus to interfere with the overweight body and the excessive deposit of fats by appetite repression and stimulation of the metabolic pathways and energy expenditure [54]. Decreased sensitivity and response to leptin action results in the development of leptin resistance leading to hyperleptinemia, which occurs mostly during the early stages of obesity and which is closely related to the onset of insulin resistance [55]. Total calystegines of white henbane significantly potentiated the sensitization of EMS_ASCs_ cells to insulin by modulating the expression of key genes involved in glucose metabolism, by especially increasing the GLUT4 receptor as well as the insulin receptor substrate (IRS) transcripts; in addition, these polyhydroxylated alkaloids have also remarkably improved lipid metabolism by upregulating the expression of both adiponectin and PPAR-γ and reducing the transcription of the *leptin* gene. Several investigations have demonstrated the beneficial effects of different iminosugars on insulin resistance as well as in hyperglycemia seen in *ob/ob* mice, via a mechanism that does not require a reduction in food intake or loss of body weight. These substances appeared to have the ability to promote GLUT4 receptor translocation, to modulate insulin-stimulated glucose uptake, and interestingly to enhance insulin receptor autophosphorylation. DNJ also improved glucose tolerance and insulin sensitivity in *db/db* mice by restoring GLUT4 translocation and phosphorylation of IR and IRS. In addition, the application of fagomine significantly reduced serum leptin levels in obese rats [56–58].

Chronic metabolic stress induces UPR signaling and ER stress that are closely associated with pathophysiological and metabolic disorders, including obesity, type 2 diabetes, inflammation, and insulin resistance [59]. EMS_ASCs_ cells were dominated by highly extended ER stress, as evidenced by increased expression of *CHOP* and *PERK* and contrasted by a decrease in the level expression of *BiP* and *eI2Fα*. Upon accumulation of unfolded proteins in the ER lumen, the ER-stress-sensing luminal domain of PERK protein is activated by trans-autophosphorylation. This triggers the subsequent phosphorylation of the eukaryotic translation (eIF2α) initiation factor 2 alpha, resulting in an overall attenuation of protein translation and a reduced ER protein load [60]. However, activation of eIF2α modulates in the same time the translation of ATF4 (activating transcription factor-4), which in turn will recruit the effector UPR, CHOP (homologous protein C/EBPα, also known as of GADD153). In pathological contexts, prolonged overexpression of CHOP triggers apoptosis by different mechanisms, including repression of the anti-apoptotic factor Bcl-2 and induction of the calcium-mediated apoptotic pathway triggered by the CHOP transcription and ER oxidase-1α [61]. Our results indicated that calystegines have noticeably improved the status of endoplasmic reticulum in EMS_ASCs_ cells by reducing significantly its stress; this resulted in a downregulation of the two triggering factors PERK and CHOP and a modulation of the transcription of the genes encoding for BiP and eI2Fa sensors. All iminosugars are known to act as pharmacological chaperones in various metabolic disorders; indeed, these molecules can interact with misfolded and unstable mutant proteins that exhibit residual biological activity. They also can bind to the catalytic sites of the deficient enzymes or other misfolded proteins and lead to improvement of trafficking in the endoplasmic reticulum, resulting in improved function [62]. In addition, DNJ was also able to attenuate ER stress, as well as calystegines, mainly by reducing the expression of the gene encoding CHOP and ATF4, as well as phosphorylation of eI2Fα [63].

The role of autophagy, an essential lysosomal degradation process for maintaining cellular metabolic homeostasis in metabolic syndrome, has been extensively demonstrated [64]. EMS_ASCs_ cells in this study were prone to autophagy, by overexpressing mRNA of crucial autophagy effectors, namely *Beclin*, *Lamp2*, and *LC3*, when compared to healthy cells. This fact, being as an adaptive survival response to dysfunctional EMS, affected cells. Basically, autophagy involves the intervention of several conserved Atg (autophagy-related) proteins, among them are class III PI3 K Vps34, Atg6/Beclin1, Atg14, and Vps15/p150.73 complex and Atg8/MAP-LC3/GABARAP/GATE-16 complex for autophagosome initiation and elongation. Therewith, chaperone-mediated autophagy (CMA), which selectively degrades proteins bearing a particular pentapeptide motif (KFERQ) through direct translocation into the lysosome, is orchestrated by the hsc70/Lamp2A complex [65]. Our experiments revealed that calystegines enhanced the expression of autophagy-related protein Beclin1, which is necessary for the initiation of autophagosome formation during autophagy. In addition, calystegines increased the levels of *LC3* transcripts. What is more is the alkaloids significantly stimulated, in the same time, the expression of *Lamp2* gene, as well as accumulation of its protein as evidenced by immunofluorescence staining; this suggests that calystegines could also modulate the chaperone-mediated autophagy. Zhu et al. [66] have shown that the iminosugar D-lentiginosine, whose structure is closely similar to that of calystegine B2, stimulated autophagy effectively by upregulating *Beclin 1*, thus causing the conversion of LC3-I to LC3-II through an inhibition of Akt/mTOR signaling by downregulating the expression of Akt and repressing its phosphorylation; these observations could thus correlate with the fact that calystegines have not exerted any upregulation effect on the *Akt* gene expression, already repressed under the metabolic syndrome condition.

## Conclusion

It resulted from this investigation that calystegines extracted from *Hyoscyamus albus* seeds have the ability to rescue ASCs affected by metabolic syndrome, by promoting their survival through apoptosis blocking and improving the dynamics status of mitochondria. The most interesting observation of this study lies in the molecular chaperone properties of these alkaloids, evidenced during their minimization of ER stress and their modulation of autophagy for the maintenance of the cellular homeostasis. In order to develop new therapeutic strategies for the management of metabolic syndrome, it would be interesting to provide a better understanding of the molecular pathways involved in the beneficial effects of calystegines for the improvement of cellular homeostasis, and especially that of pharmacological chaperones.

## Data Availability

All datasets generated and/or analyzed during the current study are presented in the article, the accompanying source data, or supplementary information files, or are available from the corresponding author upon reasonable request.

## Abbreviations

7-AAD: 7-Aminoactinomycine D
AAPH: α,α′-Azodiisobutyramidine dihydrochloride
ABTS: 2,2′-Azino-bis3-ethylbenzothiazoline-6-sulphonic acid
ADIPOQ: Adiponectin
AKT: Protein kinase B
AMPK: 5′ AMP-activated protein kinase
ASCs: Adipose-derived stromal stem cells
ATF4: Activating transcription factor 4
Atg: Autophagy-related
BAX: Bcl-2-associated X protein
Bcl2: B cell lymphoma 2
BiP: Binding immunoglobulin protein
BrdU: Bromodeoxyuridine
cAMP: Cyclic adenosine monophosphate
Casp9: Caspase 9
CD: Cluster of differentiation
CHOP: CCAAT-enhancer-binding protein homologous protein
CMA: Chaperone-mediated autophagy
CREB: cAMP-response element-binding protein
CTCF: Corrected total cell fluorescence
DAPI: 4′,6-Diamidino-2-phenylindole
DHE: Dihydroethidium
DMEM: Dulbecco modified Eagle medium
DNJ: Deoxynojirimycin
DPPH: 2,2-Diphenyl-1-picrylhydrazyl
eIF2-α: Eukaryotic initiation factor 2
EMS: Equine metabolic syndrome
ER: Endoplasmic reticulum
FBS: Fetal bovine serum
FGF: Fibroblast growth factor
GAPDH: Glyceraldehyde 3-phosphate dehydrogenase
GC-MS: Gas chromatography–mass spectrometry
GLUT4: Glucose transporter 4
HBSS: Hank’s balanced salt solution
HGF: Hepatocyte growth factor
HRP: Horseradish peroxidase
Hsc70: Heat shock cognate 71 kDa protein
IL: Interleukin
IR: Insulin resistance
IRS: Insulin receptor substrate
IκB: Nuclear factor-κB kinase
JNK: c-Jun N-terminal kinase
LAMP2: Lysosome-associated membrane protein 2
LC3: Microtubule-associated protein 1A/1B-light chain 3
Lep: Leptin
MMP: Mitochondrial membrane potential
MPT: Mitochondrial permeability transition
MS: Mass spectrometry
MS: Metabolic syndrome
MSCs: Mesenchymal stem cells
MSTFA: N-Methyl-N-(trimethylsilyl)trifluoroacetamide
mtDNA: Mitochondrial DNA
mTOR: Mammalian target of rapamycin
MVs: Microvesicles
NO: Nitric oxide
OXPHOS: Oxidative phosphorylation
PERK: Protein kinase R (PKR)-like endoplasmic reticulum kinase
PFA: Paraformaldehyde
PI3K: Phosphoinositide-3-kinase–protein kinase
PKCs: Protein kinases C
PPARγ: Peroxisome proliferator-activated receptor γ
PS: Penicillin and streptomycin
RNS: Reactive nitrogen species
ROS: Reactive oxygen species
RT-qPCR: Quantitative reverse transcription polymerase chain reaction
SEM: Scanning electron microscopy
Smac/DIABLO: Second mitochondria-derived activator of caspase/ Direct Inhibitor of Apoptosis-Binding protein with LOw pI
SREBP1C: Sterol regulatory element-binding protein 1
TNFα: Tumor necrosis factor α
VEGF: Vascular endothelial growth factor
Vps: Vacuolar protein sorting-associated protein
